# Effect of single and combined median nerve stimulation and repetitive transcranial magnetic stimulation in patients with prolonged disorders of consciousness: a prospective, randomized, single-blinded, controlled trial

**DOI:** 10.3389/fnagi.2023.1112768

**Published:** 2023-04-24

**Authors:** Qi Xiong, Kai Le, Yunliang Tang, Wen Ye, Yan Wang, Yuan Zhong, Yao Zhou, Zhen Feng

**Affiliations:** Department of Rehabilitation Medicine, The First Affiliated Hospital of Nanchang University, Nanchang, Jiangxi, China

**Keywords:** disorders of consciousness, median nerve stimulation, repetitive transcranial magnetic stimulation, clinical trial, brain injury

## Abstract

**Objective:**

To investigate the efficacy of median nerve stimulation (MNS) combined with repetitive transcranial magnetic stimulation (rTMS), MNS alone, and rTMS alone in elevating the level of consciousness in patients with prolonged disorders of consciousness (pDOC).

**Participants and methods:**

We enrolled 75 eligible inpatients suffering from pDOC as a result of traumatic or non-traumatic brain injury. Participants were randomly assigned to one of the following three treatment groups: (1) rTMS+sham-MNS; (2) MNS + sham-rTMS; or (3) MNS + rTMS. The rTMS protocol involved stimulation above the left dorsolateral prefrontal cortex at a 10 Hz frequency and 90% resting motor threshold. The MNS protocol involved the delivery of a 15–20 mA current at the median nerve point 2 cm from the wrist crease of the right distal forearm. The primary outcome was the change from baseline of the Coma Recovery Scale-Revised (CRS-R) score after treatment. Secondary outcomes included post-treatment changes from baseline of the Glasgow Coma Scale (GCS) score, awaken ratio, electroencephalography (EEG) scores, and the latency and amplitude of N20 on somatosensory evoked potentials.

**Results:**

Before the intervention, there were no significant differences between groups in the CRS-R, GCS scores, age, duration of pDOC, clinical diagnosis, EEG scores, latency and amplitude of N20, sex, job, marital status, education level, or disease etiology. Within the three groups, the total CRS-R, GCS scores and amplitude of N20 on both side significantly increased and latency of N20 on poor side significantly decreased post-intervention. Significantly greater improvement in CRS-R, GCS total scores, amplitude of N20 on both side and latency of N20 on the poor side were observed in the MNS + TMS group compared to those of the groups receiving rTMS alone or MNS alone. The patients receiving TMS and MNS intervention showed a greater EEG activity improvement, and the EEG activity improved ratio significantly differ between groups, while there were no significant differences in the awakening ratios between the three groups.

**Conclusion:**

The combination of MNS + rTMS was more efficacious in improving the level of consciousness than MNS alone or rTMS alone in patients with pDOC.

## 1. Introduction

Approximately 10–15% of individuals develop disorders of consciousness (DOC) following severe acquired brain injuries or nervous system dysfunction (Andriessen et al., 2011). There are two essential elements of consciousness: arousal and content. Disruption one or both of these elements may result in DOC. Prolonged disorder of consciousness (pDOC) are defined by a coma condition usually lasting more than 28 days. In the United States, approximately 300,000 patients suffer from prolonged DOC (pDOC) (Giacino et al., 2018), whereas in Europe, the prevalence has been reported to be as high as 6.1 per 100,000 individuals (van Erp et al., 2014). In China, at least 300,000 to 500,000 patients have been diagnosed with DOC, with more than 70,000 new patients being diagnosed every year, resulting in an annual cumulative medical expenditure of 30 to 50 billion ￥ (Chen et al., 2020). DOC always leads to severe complications, including but not limited to pneumonia, digestive tract hemorrhage, and liver dysfunction, poor clinical outcomes, and high mortality rates. Within a year after experiencing DOC, 35% of patients will die, and only 40% will experience an improved level of consciousness (Nekrasova et al., 2021).

Many studies (Thibaut et al., 2019) have investigated means of elevating the level of consciousness in patients with DOC through pharmacological treatments (e.g., amantadine, zolpidem, or ziconotide), sensory stimulation (tactile, auditory, visual, gustatory, proprioceptive, or olfactory), musical therapy, electrical stimulation (invasive or non-invasive brain stimulation), hyperbaric oxygen therapy, acupuncture, massage, and traditional Chinese medicine-based therapies. Although there are several treatment options available for patients suffering from DOC, all have demonstrated limited efficacy.

There are currently no universally accepted treatments for increasing the level of consciousness among patients experiencing DOC as a result of traumatic or non-traumatic brain injury. Non-invasive neuromodulatory techniques (Feng et al., 2020) such as median nerve stimulation (MNS), repetitive transcranial magnetic stimulation (rTMS), transcranial direct current stimulation, and vagus nerve stimulation have emerged as potentially powerful tools to enhance the level of consciousness in patients with DOC, as assessed be behavioral assessments and other means.

MNS has been widely utilized in clinical practice as a promising wake-promoting treatment in patients with DOC for almost two decades since its effects were first reported in Yokohama et al. (1996). Several previous studies have shown that MNS can improve the level of consciousness in comatose patients following brain injury. Liu et al. (2008b) reported that MNS can influence the consciousness of patients in a comatose state and increase cerebral blood flow. Similarly, a randomized controlled study conducted by Nekkanti et al. (2016) demonstrated that patients with DOC regained consciousness after receiving 4 weeks of MNS treatment.

Over the past 10 years, rTMS has also been widely used in clinical practice to help patients with pDOC regain consciousness (Xia et al., 2018); however, the frequency parameters selected in the rTMS protocol differentially affect cortical excitability. Low frequency rTMS (≤1 Hz) reduces cortical excitability, whereas high frequency rTMS (≥5–20 Hz) increases it (Chervyakov et al., 2015). Naro et al. (2015) found that rTMS of 10 Hz applied over the dorsolateral prefrontal cortex can elevate the level of consciousness and restore connectivity within several cortical areas in patients in a vegetative state (*VS*), and He et al. (2018) found that rTMS applied at a frequency of 20 Hz induced long-lasting behavioral improvements along with significant changes in the electroencephalography (EEG) power spectra. A study of resting-state EEG conducted by Bai et al. (2016) found that rTMS can significantly increase the TMS-evoked potential perturbation complexity index in patients with DOC.

Collectively, these results suggest that MNS and rTMS are efficacious in improving the level of consciousness in such patients. However, to the best of our knowledge, no studies have investigated the effects of concomitant treatment with both rTMS and MNS in patients with DOC. Therefore, this study aimed to evaluate whether synergistic effects can be induced by combining central and peripheral neuromodulatory interventions through a randomized, single-blinded, controlled study by comparing the outcomes of patients receiving MNS + rTMS, MNS + sham-rTMS, and rTMS+sham-MNS as add-ons to routine interventions (i.e., pharmacotherapy, acupuncture, or hyperbaric oxygen).

In this study, our main hypothesis was that the combination of MNS and rTMS would improve the level of consciousness in patients with DOC compared to the effects of either MNS alone or rTMS alone.

## 2. Materials and methods

### 2.1. Study design

This clinical study was designed as a prospective, single-blinded, randomized, controlled trial, conducted at a single-center. The study was approved by the Ethics Committee of the First Affiliated Hospital of Nanchang University (No. 2020–061-2). Written informed consent was obtained from the legal guardians or family members of all patients. The protocol was registered in the Chinese Clinical Trial Registry (Registration number: ChiCTR2100043784).

### 2.2. Participants and recruitment

The participants were recruited from the neurorehabilitation unit of the First Affiliated Hospital of Nanchang University from January 2021 to June 2022. The inclusion criteria were as follows: (1) clinical diagnosis of a vegetative state/unresponsive wakefulness syndrome (*VS*/UWS) or minimally conscious state (MCS), according to the standard criteria (Giacino et al., 2002; Laureys et al., 2010); (2) age ≥ 18 years; (3) pDOC with a traumatic, vascular, or anoxic etiology; (4) written informed consent for inclusion from each patient’s family members. The exclusion criteria were as follows: (1) a critical illness and unstable vital signs; (2) active cerebral hemorrhage; (3) the presence of metal objects in the brain; (4) the presence of a pacemaker; (5) unconsciousness resulting from other causes, such as that accompanied by intracranial infection; (6) epileptic seizures experienced within 1 month prior to enrolment which is a relative contraindication for rTMS treatment; and (7) pregnancy.

### 2.3. Randomization and blinding

Block randomization was used to allocate the participants at a 1:1:1 ratio to one of the following three groups: (1) the rTMS+sham-MNS group; (2) the MNS + sham-rTMS group; and (3) the MNS + rTMS group. An independent statistician from a center of evidence-based medicine generated and verified the random allocation sequence using the “plan” function of SAS software version 9.03 (SAS Institute, Inc), with a random block size of six. The random allocation sequences were concealed from the caregivers and therapists, as well as the physicians who were responsible for the patient management and behavioral assessments. The randomization table was concealed in a locked cabinet and was accessible only to the researcher performing the stimulation protocol. The patients were blinded to the stimulation protocol due to their loss of consciousness.

### 2.4. Interventions

All participants in the three groups received conventional rehabilitation including pharmacotherapy, acupuncture, and hyperbaric oxygen intervention.

#### 2.4.1. Right MNS

The right forearm was placed in a supine position, and the median nerve around the wrist was located. The skin was wiped with an alcohol swab to facilitate contact and reduce skin resistance. A 7 cm × 5 cm rubber electrode was placed on the skin to deliver the electrical stimulation. The active stimulating electrode was placed 2 cm from the volar aspect of the right distal forearm over the median nerve, and the inactive electrode was affixed to the right thenar muscle. All electrodes were secured with adhesive tape. A neuromuscular electrical stimulator (Nuocheng Electric Ltd., Jiangxi, China) provided asymmetric biphasic pulse trains at a current amplitude of 15 to 20 mA (depending on the patient’s tolerance), a pulse width of 300 μs, and a frequency of 40 Hz. The stimulation was on for 20 s and off for 40 s. A slight contraction of the patient’s right hand after stimulation indicated that the intensity of the stimulation was satisfactory. The stimulation was performed for 8 h per day. Treatment was administered six times per week for 4 weeks.

#### 2.4.2. Sham MNS

The anode of the electrode was placed on the right distal forearm over the median nerve without stimulation delivered.

#### 2.4.3. rTMS

A Magstim R2 stimulator delivered the rTMS pulses with a figure-eight focusing coil (Magstim Co. Ltd., Whitland, United Kingdom). The coil was placed tangentially toward the scalp over the left dorsolateral prefrontal cortex (10/20 International EEG system position F3) for active stimulation. One daily rTMS intervention consisted of 1,000 pulses (10 Hz, 10 s). The trains were repeated 10 times, and the interval between trains was 60 s. The total time was 11 min 40 s at 90% of the resting motor threshold, defined as the minimum TMS intensity capable of evoking a muscle contraction resulting in at least five out of 10 surface electromyographic amplitudes larger than 50 μV peak-to-peak in the relaxed first dorsal interosseous muscle of the right hand. All patients assigned to a rTMS protocol received 10 sessions of rTMS six times per week for four consecutive weeks.

#### 2.4.4. Sham rTMS

The coil was tilted 90° off the scalp to ensure the absence of a magnetic field output in the subsequent intervention stage.

### 2.5. Outcomes

#### 2.5.1. Primary outcome

The pre- to post-intervention change (delta) in the Coma Recovery Scale-Revised (CRS-R) score was the primary outcome measure. The CRS-R assesses 23 items across six subscales, including visual, auditory, motor, oromotor, communication, and arousal functions (Giacino et al., 2004). The higher the total score, the higher the level of consciousness. CRS-R assessments were conducted at baseline and after treatment (at least five times a week) (Wannez et al., 2017) by an independent assessor who was blinded to the stimulation interventions.

#### 2.5.2. Secondary outcomes

##### 2.5.2.1. Delta GCS total scores

The pre- to post-intervention change (delta) in the Glasgow Coma Scale (GCS) score was assessed. The GCS assesses 15 items grouped into three subscales representing motor, eye, and verbal responses to determine the level of consciousness (Kebapçı et al., 2020). The GCS scores were measured by the same assessor at study entry and at the end of treatment.

##### 2.5.2.2. The awakening ratio

An improvement in the clinical diagnosis, as determined by repeated CRS-R assessments, represents the gold standard for the diagnosis of pDOC (Wannez et al., 2017). *VS*/UWS is defined as a condition in which patients open their eyes but show no clinical evidence of consciousness (Laureys et al., 2010), and the most frequent signs of consciousness in MCS patients include fixation, visual pursuit, localization to noxious stimuli, reproducible movements in response to commands, and automatic motor responses, MCS minus (MCS−) patients show only lower level non-reflexive function, while MCS plus (MCS+) patients demonstrate more complex or purposeful behaviors, such as command following or intelligible verbalisation (Giacino et al., 2002). Emergence from an MCS is defined as an improvement in consciousness characterized by the ability to communicate precisely with others (i.e., family members or doctors) or being able to use objects (Machado et al., 2010). The *VS*/UWS state is the lowest level of consciousness, whereas emergence from MCS (EMCS) represents the highest level of consciousness in patients with DOC. The outcome was classified as “improved” if there was an improvement in the clinical diagnosis at the end of treatment compared to the diagnosis at the time of study entry (e.g., a patient classified as *VS*/UWS at study entry who improved to MCS−, MCS+, or emerged from MCS, or a patient classified as MCS− at study entry who experienced an elevation to MCS+ or emerged from MCS−). The clinical outcome was classified as “not improved” if the clinical diagnosis at the end of treatment was not an improvement over that at the time of study entry (i.e., a patient classified as *VS*/UWS at study entry whose state persisted, or a patient classified as MCS− at study entry who experienced a worsening to *VS*/UWS or maintained an MCS− status). The awakening ratio was calculated based on the number of participants whose diagnosis improved divided by the total number of participants.

##### 2.5.2.3. EEG activity improved ratio

EEG recordings were conducted using Sierra Wave software (Cadwell Laboratories, Inc.). The 16-lead disc electrodes were placed according to the international 10/20 system standard, and the grading criteria followed Young’s grading scheme for DOC (Young et al., 1997). Grade I patients had delta/theta waves >50% of those recorded (not theta coma). Grade II patients exhibited triphasic waves. Grade III patients demonstrated burst-suppression. Grade IV patients showed alpha/theta/spindle coma (unreactive). Grade V patients exhibited epileptiform activity (no burst-suppression pattern). Grade VI patients demonstrated suppression of all EEG waves (< 20 μV). EEG scores were recorded before and after treatment. The higher the scores, the worse the EEG activity. The EEG outcome was classified as “improved” if the EEG scores at the end of treatment deceased over that at the time of study entry, if the EEG scores remained the same or increased compared that at the time of study entry, the EEG outcome were defined as “not improved.” The EEG improved ratio was calculated based on the number of participants whose EEG performance improved divided by the total number of participants.

##### 2.5.2.4. Somatosensory evoked potentials

SEPs (Somatosensory evoked potentials) were recorded using Cadwell Sierra Wave software (Cadwell, Kennewick, WA, United States). The recording electrodes were placed 2 cm posterior to C3 & C4 according to the international 10/20 system standard, and the reference electrode was positioned at Fz. Stimulation was delivered to the median nerve point 2 cm from the volar aspect of the right distal forearm. Three SEP trials were recorded and analyzed. The bilateral N20 latencies (ms) and amplitudes (μV) were measured before and after treatment (if the damaged side was on the left, that was defined as the “poor” side and right side was defined as the “good” side). For example, a patient with left thalamic hemorrhage after stoke, then left side is poor side and right side is good. The delta latency and amplitude of N20 on both sides were defined as the pre- to post-intervention change in latency and amplitude of N20.

### 2.6. Study power and sample size calculation

The required sample size was calculated based on delta CRS-R total scores, the delta CRS-R scores after rTMS stimulation is 3.0 ± 2.0 according to a previous study by Ge et al. (2021). A previous study in our coma group determined that MNS can result in a 2.7 ± 1.7-point improvement in CRS-R scores. In addition, our pilot trial found the patients who underwent rTMS+MNS experienced a 5.5 ± 4.2-point increase in CRS-R scores. The required sample size was calculated based on one-way analysis of variance (ANOVA) F tests using PASS software (PASS 15.0, NSCC, LLC, United States). The formula is:


N=φ2(∑i=1kSi2/k)[∑i=1k(Xi−X)2/(k−1)]

S_i_ and X_i_ is the standard deviation and mean of delta CRS-R scores, X= $\overset{k}{\underset{i=1}{\sum }}X_{i}/k$ , k = 3. With a type I error rate of 5% (*α* = 0.05) and 80% power (*β* = 0.20), the mean delta CRS-R scores in the three groups were 3.0, 2.7, and 5.5, respectively, with standard deviations of 2.0, 1.7, and 4.2, respectively. The estimated required sample size was 21 participants per group. Allowing for a 20% dropout rate during the study, a minimum total of 75 participants were needed to reach the target of 25 participants per group.

### 2.7. Statistical analysis

Continuous variables are expressed as the mean ± standard deviation, and categorical variables are expressed as counts and/or frequencies. The Shapiro–Wilk test was used to assess the normality of continuous variables. As the duration of pDOC departed significantly from the norm, the Kruskal-Wallis test was used to compare that variable between the three groups. Because the baseline and delta CRS-R, and GCS scores, and the latency and amplitude of N20 were normally distributed, these variables were compared between groups by ANOVA. Paired *t*-tests were used to analyze differences in CRS-R, GCS scores, latency and amplitude of N20 within groups before and after treatment. Baseline characteristics were compared between groups using Pearson’s chi-square tests for categorical variables (such as the awakening ratio, EEG activity, job, disease etiology, sex, marital status, and education level). All analyses were performed using the statistical package R (The R Foundation).^1^ A two-sided *p* value <0.05 was considered statistically significant.

## 3. Results

### 3.1. Description of the sample

**Figure 1:**
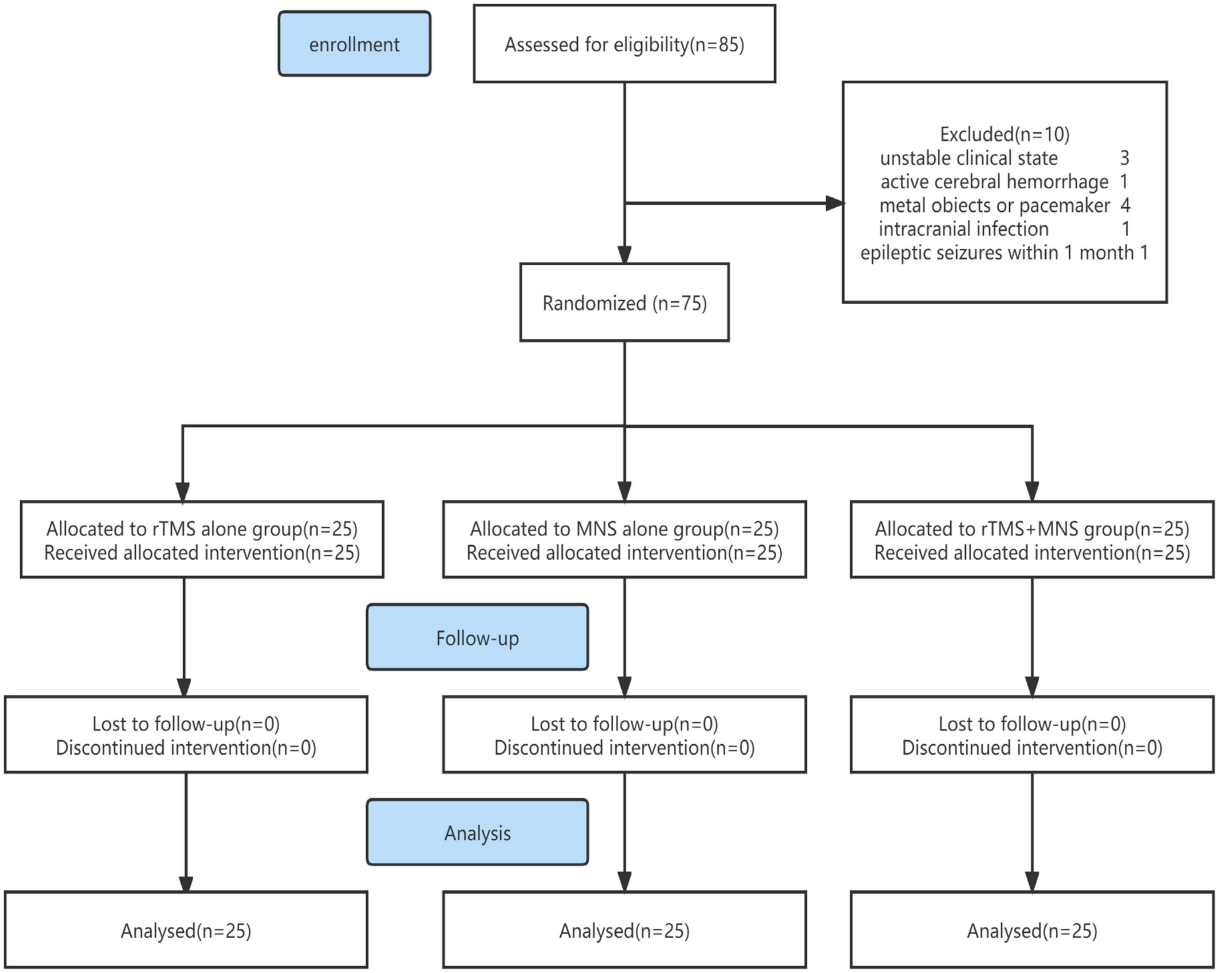
Study flowchart.

The study flowchart is presented in Figure 1. The demographic and basic characteristics of the three groups are presented in Table 1. Out of the 85 patients who were screened for this study, 75 patients with pDOC (mean age: 57.13 ± 14.96 years) fulfilled the selection criteria, 34 of whom met the *VS* criteria and 41 of whom met the MCS criteria. The mean duration of pDOC was 61.53 ± 50.58 days, the mean CRS-R score was 6.35 ± 2.24, and the mean GCS score was 7.51 ± 1.81. Twenty-eight (37.3%) participants were women, and 38 (50.7%) had a condition with a vascular etiology. There were 63 (84%) participants with a pDOC duration ranging from 28 to 90 days. No participants were lost to follow-up, and none discontinued the intervention. There were no significant differences between the three groups in terms of the baseline CRS-R, GCS, EEG scores, amplitude and latency of N20, age, duration of pDOC, clinical diagnosis, sex, job, marital state, education level, or etiology.

**Table 1:** Demographic and clinical characteristics of pDOC patients in the three groups.

Groups	rTMS	MNS	MNS + rTMS	value of *p*
*N*	25	25	25	
Age (years)	58.76 ± 13.79	58.76 ± 12.94	53.96 ± 18.97	0.45
Duration of DOC (days)	62.08 ± 41.50	57.0 ± 46.13	65.52 ± 57.85	0.44
28–90	21 (84.00%)	23 (92.00%)	19 (76.00%)	
61–180	3 (12.00%)	1 (4.00%)	5 (20.00%)	
>180	1 (4.00%)	1 (4.00%)	1 (4.00%)	
CRS-R	6.12 ± 2.49	6.76 ± 1.79	6.16 ± 2.21	0.51
GCS	7.36 ± 1.91	7.76 ± 1.67	7.40 ± 1.68	0.67
Latency of N20 on good side (ms)	20.57 ± 1.20	21.17 ± 1.93	20.77 ± 1.27	0.36
Latency of N20 on poor side (ms)	25.36 ± 3.23	25.93 ± 4.27	25.73 ± 2.35	0.85
Amplitude of N20 on good side (μV)	1.74 ± 1.13	1.95 ± 1.51	2.04 ± 1.57	0.73
Amplitude of N20 on poor side (μV)	1.03 ± 0.96	1.02 ± 1.28	0.78 ± 0.61	0.66
**Diagnosis**				0.94
*VS*	12 (48.00%)	11 (44.00%)	11 (44.00%)	
MCS	13 (52.00%)	14 (56.00%)	14 (56.00%)	
**Sex**				0.40
female	12 (48.00%)	8 (32.00%)	8 (32.00%)	
male	13 (52.00%)	17 (68.00%)	17 (68.00%)	
**Job**				0.06
No	9 (36.00%)	2 (8.00%)	7 (28.00%)	
Yes	16 (64.00%)	23 (92.00%)	18 (72.00%)	
**Married**				0.08
No	1 (4.00%)	1 (4.00%)	5 (20.00%)	
Yes	24 (96.00%)	24 (96.00%)	20 (80.00%)	
**Education**				0.17
Primary	14 (56.00%)	14 (56.00%)	8 (32.00%)	
Middle	7 (28.00%)	9 (36.00%)	15 (60.00%)	
High	4 (16.00%)	2 (8.00%)	2 (8.00%)	
**Etiology**				0.27
Traumatic	8 (32.00%)	12 (48.00%)	8 (32.00%)	
Vascular	12 (48.00%)	11 (44.00%)	15 (60.00%)	
Anoxia	5 (20.00%)	2 (8.00%)	2 (8.00%)	

DOC, disorders of consciousness; CRS-R, Coma Recovery Scale-Revised; GCS, Glasgow Coma Scale; VS, vegetative state; MCS, minimally conscious state.

### 3.2. CRS-R total scores

**Figure 2:**
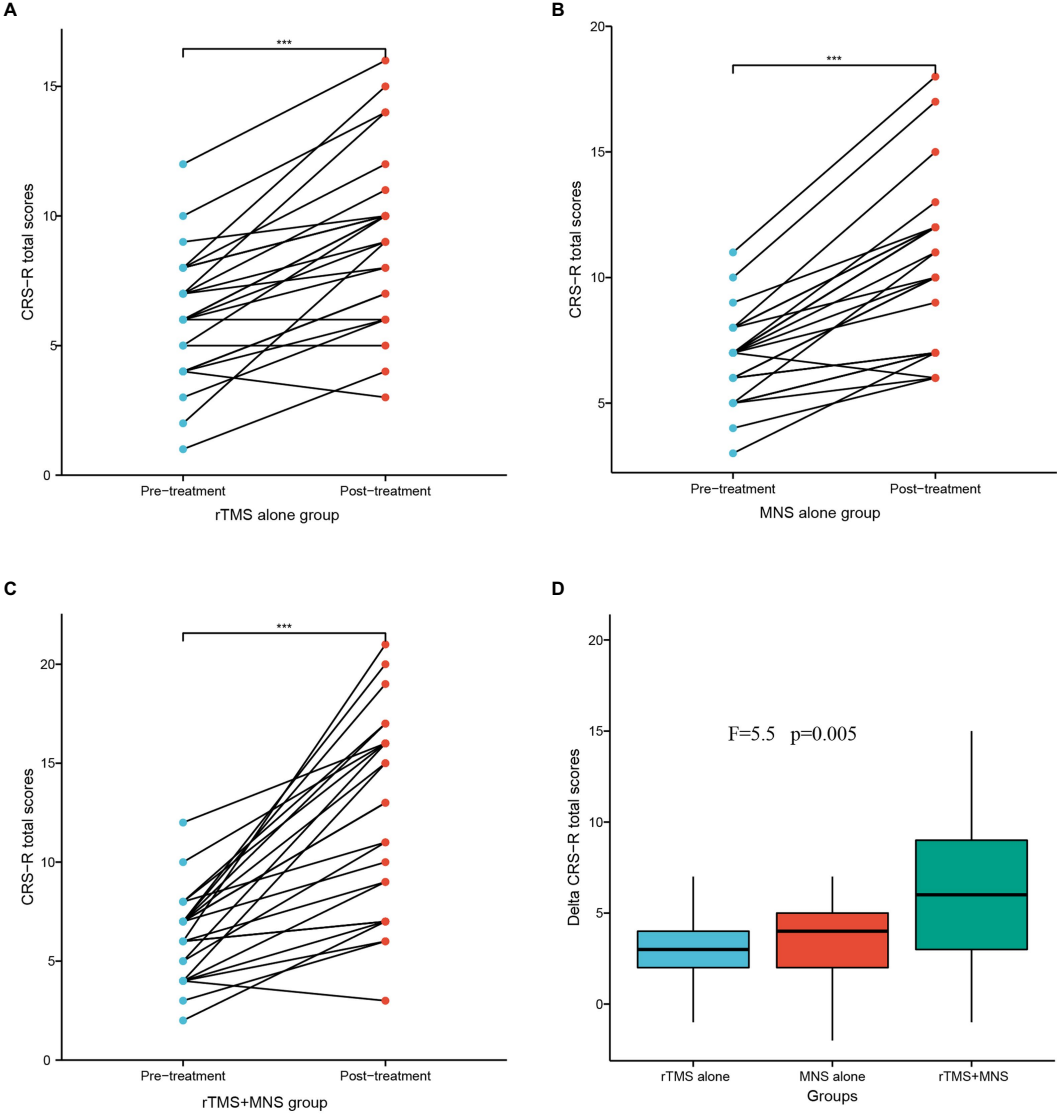
CRS-R total scores following 4 weeks of treatment for the rTMS alone, MNS alone and rTMS+MNS groups. A significant increase in the CRS-R scores compared with pre-treatment in the **(A)** rTMS alone, **(B)** MNS alone and **(C)** rTMS+MNS. **(D)** The changes in CRS-R scores between the three groups were statistically significant. ***means *p* < 0.001.

The baseline, post-treatment, and delta CRS-R total scores were normally distributed and were analyzed by paired *t*-tests within groups. The delta CRS-R scores were analyzed by ANOVA between groups. The results of these analyses are shown in Figure 2. Within the three groups, the total CRS-R scores were significantly increased post-intervention compared with the respective values measured before treatment (Figures 2A–C). The between-group comparisons revealed that the delta CRS-R scores in the MNS + TMS group were significantly higher than those in the groups receiving TMS alone or MNS alone (Figure 2D). The mean delta CRS-R scores in the three groups were 3.48 ± 2.45, 3.56 ± 2.22, and 6.12 ± 4.16 in the three groups, respectively.

### 3.3. GCS total scores

**Figure 3:**
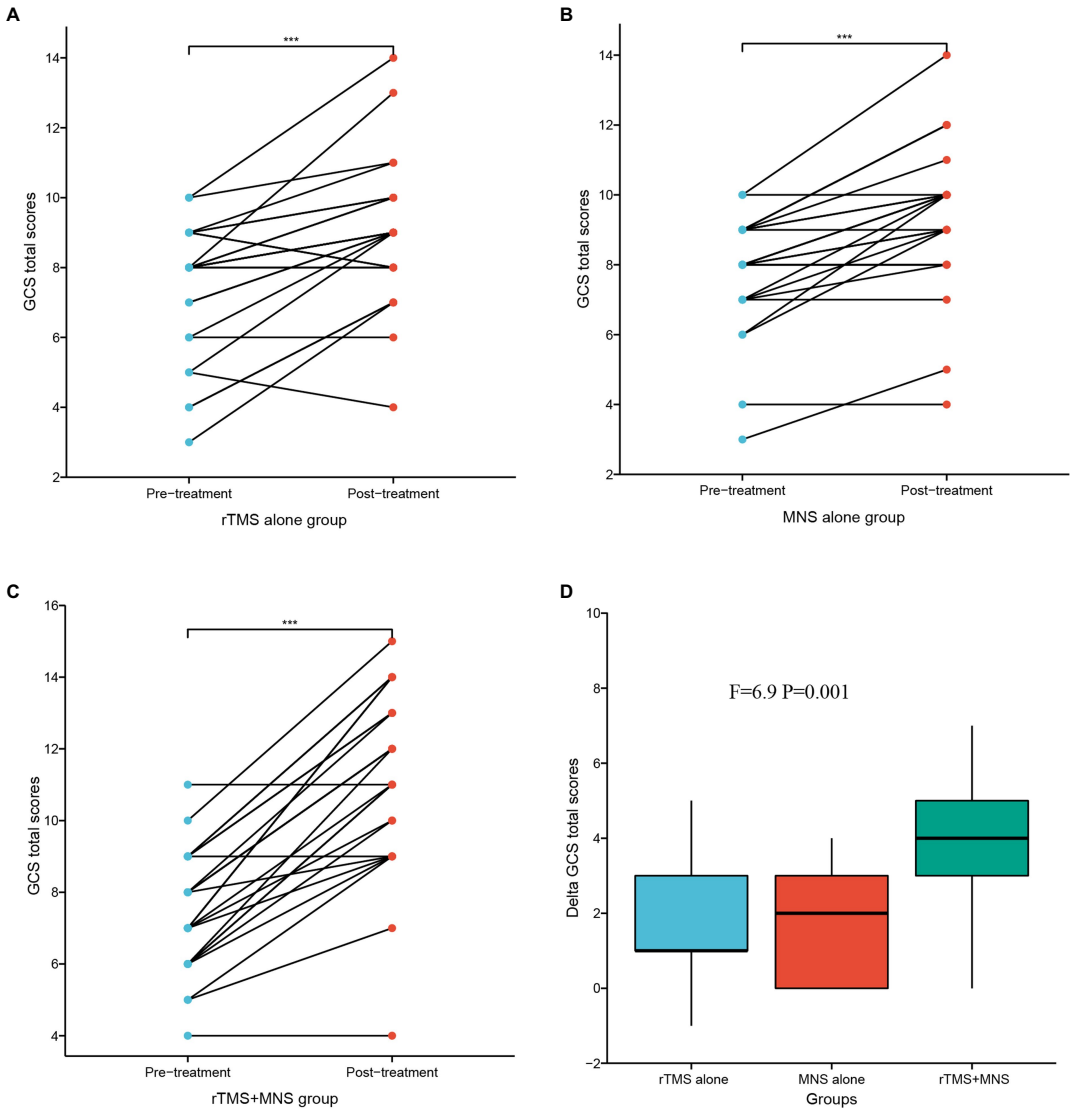
GCS total scores following 4 weeks of treatment for the rTMS alone, MNS alone and rTMS+MNS groups. A significant increase in the GCS scores compared with pre-treatment in the **(A)** rTMS alone, **(B)** MNS alone and **(C)** rTMS+MNS. **(D)** The changes in GCS scores between the three groups were statistically significant. ***means *p* < 0.001.

Paired *t*-tests were used to analyze the differences in the GCS total scores within groups before and after intervention, and ANOVA tests were used to compare the differences in the GCS total scores between the three groups. The results of these analyses are shown in Figure 3. There were significant increases in the GCS total scores after intervention compared with the baseline values in the three groups (Figures 3A–C). Significantly higher delta GCS scores were observed in the MNS + TMS group compared to those of the groups receiving rTMS alone or MNS alone (Figure 3D). The mean delta GCS scores in the three groups were 1.60 ± 1.66, 1.65 ± 1.32, and 3.72 ± 1.97, respectively.

### 3.4. The awakening ratio

The clinical diagnoses before and after treatment and the improvements in consciousness level in the three groups are presented in Table 2. In the group receiving TMS alone, 12 (48%) participants exhibited an improvement in their clinical diagnosis, including four of the 12 patients with a *VS* diagnosis at entry who were elevated to MCS− or MCS+ and eight of the 13 individuals with a MCS− diagnosis at entry who were improved to MCS+ or EMCS. Among all the patients in the group receiving MNS alone, 13 (52%) experienced an improved outcome in terms of the clinical diagnosis. Five of 11 patients with a baseline *VS* diagnosis had an improved diagnosis of MCS− or MCS+ post-intervention, and only 5/14 participants with an MCS− diagnosis at entry had the same diagnosis post-intervention. In addition, our results demonstrated that 6/11(54.5%) patients with a *VS* diagnosis before treatment retained their *VS* status post-intervention in the MNS + TMS group, whereas 12/14(85.7%) patients with an MCS− diagnosis had an improved diagnosis of MCS+ or EMCS post-treatment. The awakening ratio did not significantly differ between groups, with 68% participants in the TMS + MNS group showing improvement, compared with 48 and 52% in the groups receiving TMS alone or MNS alone (Table 3).

**Table 2:** Clinical diagnosis pre- and post-treatment and the improvements in consciousness level in the three groups.

Patient	TMS alone			MNS alone			TMS + MNS		
	Pre	Post	Outcome	Pre	Post	Outcome	Pre	Post	Outcome
P1	*VS*	*VS*	Persistency	MCS−	MCS+	Improved	MCS−	MCS+	Improved
P2	MCS−	MCS−	Persistency	MCS−	EMCS	Improved	MCS−	EMCS	Improved
P3	MCS−	MCS+	Improved	*VS*	MCS−	Improved	*VS*	MCS−	Improved
P4	MCS−	MCS−	Persistency	*VS*	MCS−	Improved	*VS*	MCS−	Improved
P5	*VS*	*VS*	Persistency	*VS*	*VS*	Persistency	MCS−	MCS+	Improved
P6	MCS−	MCS+	Improved	*VS*	*VS*	Persistency	*VS*	*VS*	Persistency
P7	*VS*	*VS*	Persistency	MCS−	MCS−	Persistency	*VS*	*VS*	Persistency
P8	MCS−	MCS+	Improved	MCS−	MCS+	Improved	MCS−	MCS+	Improved
P9	*VS*	MCS−	Improved	MCS−	MCS+	Improved	MCS−	MCS−	Persistency
P10	MCS−	MCS−	Persistency	*VS*	MCS−	Improved	MCS−	MCS+	Improved
P11	MCS−	MCS−	Persistency	MCS−	MCS+	Improved	MCS−	MCS+	Improved
P12	*VS*	*VS*	Persistency	MCS−	MCS+	Improved	MCS−	EMCS	Improved
P13	MCS−	MCS+	Improved	MCS−	EMCS	Improved	*VS*	*VS*	Persistency
P14	*VS*	*VS*	Persistency	MCS−	MCS−	Persistency	*VS*	MCS−	Improved
P15	*VS*	*VS*	Persistency	MCS−	MCS+	Improved	*VS*	MCS−	Improved
P16	MCS−	MCS+	Improved	MCS−	MCS−	Persistency	*VS*	MCS+	Improved
P17	MCS−	MCS+	Improved	MCS−	MCS−	Persistency	*VS*	*VS*	Persistency
P18	*VS*	MCS+	Improved	*VS*	*VS*	Persistency	MCS−	EMCS	Improved
P19	MCS−	MCS+	Improved	*VS*	*VS*	Persistency	MCS−	MCS+	Improved
P20	MCS−	EMCS	Improved	*VS*	*VS*	Persistency	MCS−	EMCS	Improved
P21	*VS*	MCS−	Improved	*VS*	*VS*	Persistency	*VS*	*VS*	Persistency
P22	MCS−	MCS−	Persistency	*VS*	MCS−	Improved	MCS−	MCS+	Improved
P23	*VS*	*VS*	Persistency	MCS−	MCS−	Persistency	MCS−	EMCS	Improved
P24	*VS*	*VS*	Persistency	MCS−	MCS+	Improved	MCS−	*VS*	Worsen
P25	*VS*	MCS−	Improved	*VS*	MCS−	Improved	*VS*	*VS*	Persistency

VS, vegetative state; MCS−, minimally conscious state minus; MCS+, minimally conscious state plus; EMCS, emergence from minimally conscious state.

**Table 3:** Comparison of electroencephalogram (EEG) activity improvement among the three groups of patients.

Groups	*n*	Not improved	Improved
rTMS	25	8 (32%)	17 (68%)
MNS	25	9 (36%)	16 (64%)
rTMS+MNS	25	4 (16%)	21 (84%)
Chi-square tests			25.6
*P* value			0.007

### 3.5. EEG activity

After the intervention at the end of 1 month, in the group of TMS alone, 17 (68%) participants showed an improvement in their EEG activity. Among all the patients in the group receiving MNS alone, 16 (64%) exhibited an improved outcome in terms of the EEG scores. Moreover, our results demonstrated that 21(84%) patients experienced an improved EEG performance post-treatment in the MNS + TMS group. The EEG activity improved ratio significantly differ between groups, with 84% participants in the TMS + MNS group showing improvement, compared with 68 and 64% in the groups receiving TMS alone or MNS alone, and the patients receiving TMS and MNS intervention show a greater EEG activity improvement.

### 3.6. SEPs

**Figure 4:**
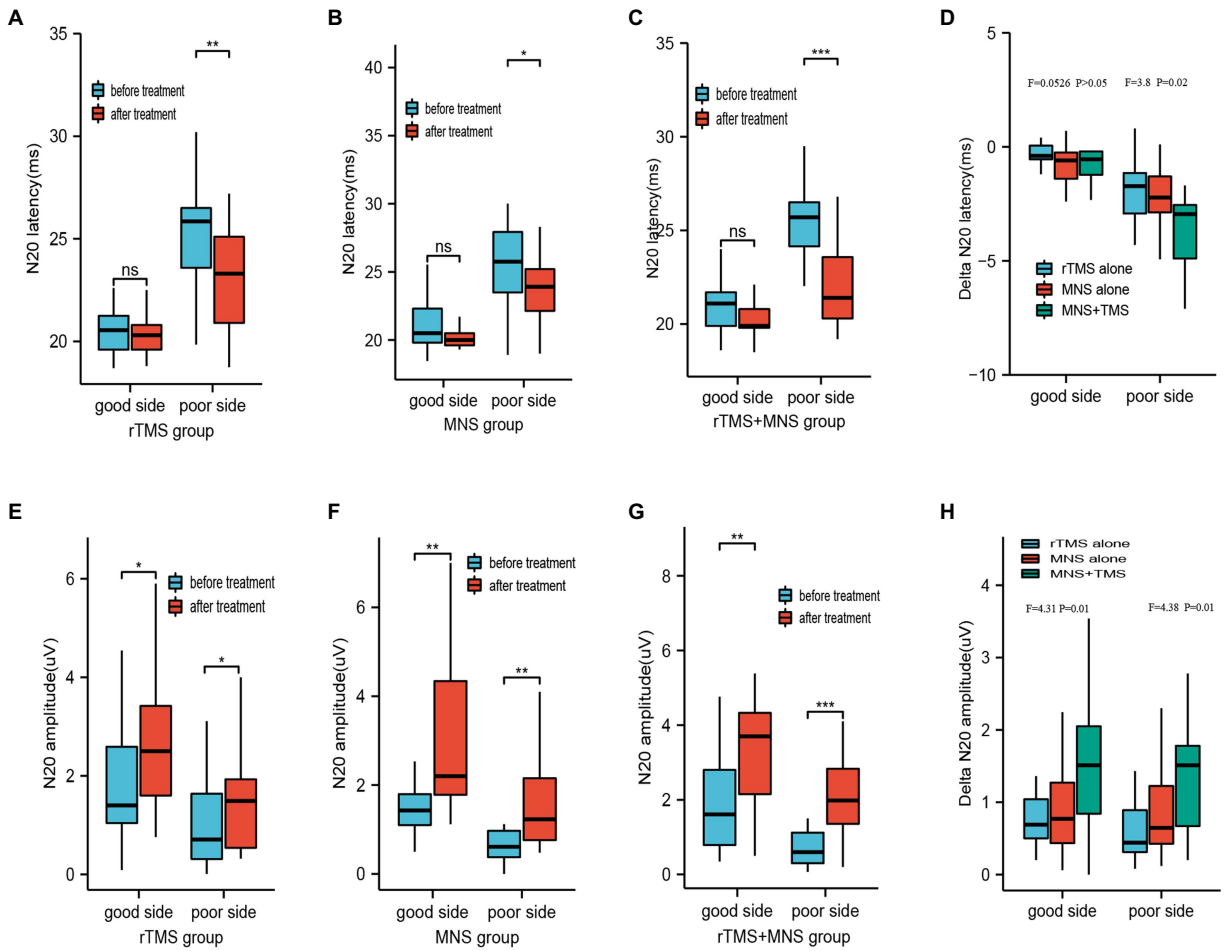
Latency and amplitude of N20 following 4 weeks of treatment for the rTMS alone, MNS alone and rTMS+MNS groups. A significant decrease in the latency of N20 on the poor sides after intervention compared with the baseline values in the **(A)** rTMS alone, **(B)** MNS alone and **(C)** rTMS+MNS, whereas the decrease measured on the “good” side were not. **(D)** The changes in latency of N20 on poor side between the three groups were statistically significant. No statistically significant changes in latency of N20 on good side between the three groups were showed. A significant increase in the amplitude of N20 on the both side compared with pre-treatment in the **(E)** rTMS alone, **(F)** MNS alone and **(G)** rTMS+MNS. **(H)** The changes in amplitude of N20 on both side between the three groups were statistically significant. rTMS, repetitive transcranial magnetic stimulation; MNS, median nerve stimulation; *means *p* < 0.05, **means *p* < 0.01, ***means *p* < 0.001.

Before treatment, three, three, and four patients exhibited an absence of N20 on the “poor” side in the rTMS, MNS, and MNS + rTMS groups, respectively. At the end of treatment, only two patients in MNS + rTMS group showed an absence of N20 on the “poor” side. The latencies and amplitudes on both sides did not significantly differ between the three groups. The latencies of N20 on the “good” side were 20.57 ± 1.20 ms, 21.17 ± 1.93 ms, and 20.77 ± 1.27 ms in the rTMS, MNS, and rTMS+MNS groups, respectively, all of which were shorter than those measured on the “poor” sides (25.36 ± 3.23 ms, 25.93 ± 4.27 ms, and 25.73 ± 2.35 ms, respectively). There were significant decrease in the latency of N20 on the poor sides after intervention compared with the baseline values in the three groups, whereas the decrease measured on the “good” side were not (Figures 4A–C). Significantly greater delta latency of N20 on the poor side were observed in the MNS + TMS group compared to those of the groups receiving rTMS alone or MNS alone, while no statistically significant changes in latency of N20 on good side between the three groups were showed (Figure 4D). The amplitudes of N20 on the “good” side were 1.74 ± 1.13 μV, 1.95 ± 1.51 μV, and 2.04 ± 1.57 μV in the rTMS, MNS group, and rTMS+MNS groups, respectively, all of which were higher than those measured on the “poor” sides (1.03 ± 0.96 μV, 1.02 ± 1.28 μV, and 0.78 ± 0.61 μV, respectively). There were significant increases in the amplitude of N20 on both sides after intervention compared with the baseline values in the three groups (Figures 4E–G). Significantly higher delta amplitude of N20 were observed in the MNS + TMS group compared to those of the groups receiving rTMS alone or MNS alone (Figure 4H).

## 4. Discussion

pDOC is a serious functional disorder that occurs after experiencing a severe traumatic or non-traumatic brain injury, and it is always accompanied by severe complications that lead to poor clinical outcomes and high mortality rates (Kondziella et al., 2020). Moreover, the incidence of pDOC has continued to increase in recent years (Liu S. et al., 2022). Therefore, identifying optimal comprehensive neuromodulatory methods could play a crucial role in managing pDOC and helping patients regain consciousness.

MNS is a peripheral nerve electrical stimulation treatment technique (Feller et al., 2021). The right median nerve is generally selected for electrical stimulation in the treatment of comatose patients. The technique has been widely used due to its inherent advantages, which include the fact that it is non-invasive, safe, and simple to perform. A previous study conducted by Liu et al. (2008a) demonstrated that MNS stimulation can improve GCS scores by 2 points and increase regional cerebral perfusion in comatose patients. A randomized, controlled trial by Lei et al. (2015) showed that patients in groups receiving MNS experienced an elevation in GCS scores of 2.2 points, and 122 (59.8%) patients regained consciousness. Another study by Liu Y. S. et al. (2022) demonstrated a significant increase in CRS-R scores by 3.8 points and an awakening ratio of 53.8% in those treated with MNS, along with an improvement in EEG characteristics. In the present study, the GCS and CRS-R scores were improved by 1.6 and 3.65 points, respectively, and 13 (52%) patients experienced an elevation of the level of consciousness. The patients in the present study experienced a smaller increase in the GCS scores, and the awakening ratio was lower compared to those in the study by Liu et al. There are two possibilities to explain these results. First, the duration of DOC in this study (61.53 days) was longer than that in Liu’s study (48.4 days). Second, the mean age of the patients in Liu’s cohort (42.5 years) was younger than that of the participants in our study (57.13 years). As an effective and non-invasive stimulation technique, MSN can promote wakefulness through several mechanisms. The first is that the spinoreticular component of median neurons is directly involved in the ascending reticular activating system (ARAS) (Cooper et al., 2005). The ARAS is an important pathway for sensory conduction, as it transmits a variety of internal and external stimuli to the neurons of the cerebral cortex to maintain a state of wakefulness (Kwak and Chang, 2020). Electrical stimulation of the median nerve transmits excitatory impulses through the ARAS to the cerebral cortex. The latency of N20 increases, whereas the amplitude decreases after ARAS injury, with the degree of damage being related to the level of consciousness. The present study found that the amplitudes of N20 improved and the latencies decreased after MNS stimulation, which indicated that MNS could enhance the ARAS. Second, MNS stimulation can increase the level of neurotrophic factors, such as brain-derived neurotrophic factor, and neurotransmitters such as orexins. Our previous animal study found that MNS exerted wake-promoting effects in comatose rats after traumatic brain injury by upregulating orexin-A and BDNF (Feng and Du, 2016). Third, Liu et al. (2008a) found that MNS can increase cerebral blood flow in comatose patients. Finally, the ability of MNS to enhance cerebral cortex activity, reduce brain inhibition, and improve EEG activity was supported by the findings of Buitrago et al. (2004).

As a brain stimulation technique, the safety and efficacy of high frequency rTMS have been reported by several studies (Formica et al., 2021). The first study investigating the effects of rTMS in patients with DOC was published in 2009 by Louise-Bender Pape et al. (2009). A previous study by Ge et al. (2021) demonstrated that the mean delta CRS-R total scores increased by 3 points in the rTMS group, and rTMS stimulation was capable of improving the latency of motor-evoked potentials. Legostaeva et al. (2019) reported a mean increase in CRS-R total scores of 2.1 points, and a prospective single-blinded study by Xia et al. (2017) indicated that the technique resulted in a significant improvement in CRS-R scores by 2.2 points, with an awakening ratio of 56.2%. The latter study also reported an increase in the delta band in the frontal lobe. Yet another randomized, controlled, double-blinded study by Fan et al. (2022) reported a mean change in CRS-R scores of 3.3 points following rTMS, with half of all patients experiencing an improvement in their level of consciousness. In the present study, 12 (48%) patients exhibited an improvement in the clinical diagnosis at the end of treatment and an increase in CRS-R scores of 3.65 points; these findings are consistent with those of Ge et al. and Fan et al. Possible mechanisms for the awakening effects have been reported by several studies. Jang and Kwon (2020) demonstrated increases in neural tract volume in the right prefrontal lobe of the upper ARAS after rTMS treatment in a patient with stroke who was experiencing DOC. Moreover, two brain perfusion studies (Xie et al., 2011; Liu et al., 2016) demonstrated that rTMS can significantly increase regional cerebral blood flow and cerebral blood velocity in patients with pDOC, and Zhang et al. (2021) found that rTMS can improve the excitability of the cerebral cortex.

Although MNS or TMS alone can improve the level of consciousness in patients with pDOC, neurorehabilitation in such cases is a long process that is associated with great clinical challenges (Feng et al., 2020; O'Neal et al., 2021). With the increasing needs and expectations for the rehabilitation of patients suffering from pDOC as a result of brain injury, certain limitations have emerged in relation to the use of MNS or rTMS alone to promote wakefulness (Shou et al., 2021). Thus, the results of this study are novel in that we investigated the combined use of both interventions in elevating the level of consciousness of patients with pDOC compared to the effects of either intervention alone. While patients in all three groups experienced significant improvements in the behavioral assessment scores compared to those measured at baseline before treatment, the patients in the MNS + rTMS group experienced a significantly greater increase in the CRS-R and GCS total scores compared to those of the groups receiving MNS or rTMS alone. The awakening ratios were 48, 52, and 65% in the rTMS, MNS, and MNS + rTMS groups. Additionally, the EEG score as well as the N20 latencies on the poor side were significantly decreased after treatment compared to the values measured before treatment, whereas the N20 amplitudes on the both sides after treatment were significantly higher compared to baseline levels. In the patients treated with MNS + rTMS, there were greater decreases in the EEG scores and N20 latencies on the “poor” side, along with a greater increase in N20 amplitudes on both sides, indicating that rTMS combined with MNS had a significantly greater wake-promoting effect on patients with pDOC, possibly due to synergistic effects of the two techniques in terms of improving cerebral cortical excitability and ARAS transmission. The improvements in the behavioral assessment scores, EEG activity, and the N20 amplitudes in the rTMS+MNS group were superior to those experienced by the groups receiving rTMS or MNS alone, probably because MNS stimulation was delivered at the same time as the rTMS intervention, resulting in the enhancement of the wake-promoting effects of the two treatments. As a somatosensory stimulation technique, MNS could enhances the effect of rTMS on increasing the cerebral cortical excitability and the ARAS through the brainstem reticular system and thalamus, increasing the level of consciousness in patients with DOC. Therefore, we believe that MNS and rTMS synergistically enhance the level of consciousness in patients with pDOC.

There are several limitations in our study. First, due to a small sample size, treatment effect was not allowed to analysis between the different diagnostic groups. Second, the treatment period was 1 month, and no long-term follow-up results were analyzed, leaving the long-term effects of the different treatment unclear.

In conclusion, both rTMS and MNS are clinically efficacious in improving the level of consciousness, EEG activity, and N20 amplitudes, while decreasing the N20 latencies in patients with pDOC. However, the combined use of MNS + rTMS was superior to either MNS or rTMS alone in improving the level of consciousness in such patients. The two interventions could synergistically enhance the effects of either individual treatment, possibly by improving cerebral cortical excitability and SEPs.

## Data availability statement

The raw data supporting the conclusions of this article will be made available by the authors, without undue reservation.

## Ethics statement

The studies involving human participants were reviewed and approved by the Ethics Committee of the First Affiliated Hospital of Nanchang University. The patients/participants provided their written informed consent to participate in this study.

## Author contributions

QX conducted the experiment proper and prepared the manuscript. KL, YT, and WY responsible for the management of patients. YuZ and YaZ performed the repeated diagnosis and collected the data. YW performed the statistical analysis of the data. ZF secured funding for the project. All authors have read and approved the final manuscript.

## Funding

This study was funded by the Major Research Development Program of Jiangxi Province (grant no. 20202BBG72002).

## Conflict of interest

The authors declare that the research was conducted in the absence of any commercial or financial relationships that could be construed as a potential conflict of interest.

## Publisher’s note

All claims expressed in this article are solely those of the authors and do not necessarily represent those of their affiliated organizations, or those of the publisher, the editors and the reviewers. Any product that may be evaluated in this article, or claim that may be made by its manufacturer, is not guaranteed or endorsed by the publisher.

## Footnotes

^1^ http://www.R-project.org
